# Proliferation and gene expression data of rabbit Achilles tenocyte in vitro culture in the presence of high-molecular weight hyaluronic acid (1.01–1.8 MDa)

**DOI:** 10.1016/j.dib.2025.111459

**Published:** 2025-03-15

**Authors:** Iris Miescher, Petra Wolint, Christine Opelz, Jess G. Snedeker, Pietro Giovanoli, Maurizio Calcagni, Johanna Buschmann

**Affiliations:** a Division of Plastic Surgery and Hand Surgery, University Hospital Zurich, Sternwartstrasse 14, 8091 Zurich, Switzerland; b University Clinic Balgrist, Orthopaedic Biomechanics, Forchstrasse 340, 8008 Zurich, Switzerland

**Keywords:** Doubling time, high-molecular weight hyaluronic acid, Biglycan, Decorin, Aggrecan, Collagen 1A1, Collagen 1A2, Collagen 3

## Abstract

Hyaluronic acid (HA) is a natural bio macromolecule and a good bio lubricant. As it is a predominant component of the synovial fluid, the aim was to explore the effects HA on rabbit Achilles tenocytes in vitro. The data set presents proliferation assessed by the Alamar Blue® assay; and gene expression of tenocytes that were cultured either with or without supplementation of high-molecular weight HA in a concentration of 1.4 mg/mL. There was a daily medium exchange with fresh HA supplementation. On days 3, 7 and 14 the following target genes were analysed and manifold induction was calculated against the control (no HA): Biglycan, Decorin, Aggrecan, Collagen 1A1, Collagen 1A2, Collagen 3, LOX, IL-6, TNF-α, PAR-2, ALOX-15, Tenascin-C, Tenomodulin, Mohawk, α-SMA, TIMP-1, MMP-2, MMP-9, and ki67. These data might be interesting for researchers working in the tendon field.

## Graphical abstract

**Figure:**
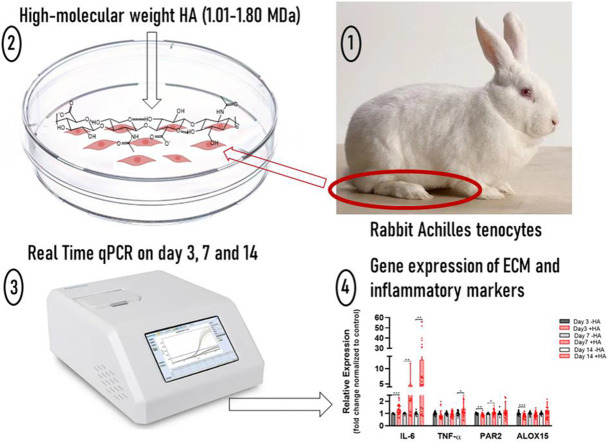

Specifications Table

**Table:** 

Subject	Genetics; cell biology
Specific subject area	Cell culture data were determined for monolayer Achilles tenocyte cultures. Tenocytes were harvested from freshly isolated New Zealand White Rabbit Achilles tendons of three rabbits. These monolayer tenocyte cultures were supplemented with high-molecular weight hyaluronic acid (1.4 mg/mL HA), and gene expression for Biglycan, Decorin, Aggrecan, Collagen 1A1, Collagen 1A2, Collagen 3, LOX, IL-6, TNF-α, PAR-2, ALOX-15, Tenascin-C, Tenomodulin, Mohawk, α-SMA, TIMP-1, MMP-2, MMP-9, and ki67 was assessed after 3, 7, and 14 days (control no HA). Moreover, proliferation data and doubling times were assessed by Alamar Blue® assay [1].
Data format	The data consist of raw analysed data.
Type of data	Excel files of the type *.xlsx* files.
Data collection	Alamar Blue® assay proliferation data: Rabbit Achilles tenocytes were cultured as monolayers either with 1.4 mg/mL HA or without HA. Cell number was assessed on days 0, 3, 5, 7, and 14, respectively. qPCR data: Rabbit Achilles tenocytes were cultured as monolayers and RNA was extracted by a RNeasy Plus Mini Kit from Qiagen, Hilden, Germany; followed by reverse transcription with the iScript Advanced cDNA Synthesis Kit (Bio-Rad, Cressier, Switzerland). The quantitative real-time PCR reactions were performed with CFX Connect Real-Time PCR Detection System (Bio-Rad, Cressier, Switzerland) and SsoAdvanced SYBR Green Supermix (Bio-Rad, Cressier, Switzerland).
Data source location	Proliferation data and gene expression data was collected in Zurich, Switzerland, at the University Hospital Zurich.
Data accessibility	**Repository name**: Mendeley Data **Data identification number**: DOI: 10.17632/2g8bmzkdnv.1 DOI: 10.17632/z4zpr8zghf.1 Direct URL to data: Rabbit Achilles tenocytes proliferation: Alamar Blue® assay data for cell cultures with or without high molecular weight hyaluronic acid having a molecular weight range of 1.01–1.8 MDa - Mendeley Data Impact of high-molecular weight hyaluronic acid on the gene expression of rabbit Achilles tenocytes in vitro - Mendeley Data
Related research article	Impact of High-Molecular-Weight Hyaluronic Acid on Gene Expression in Rabbit Achilles Tenocytes In Vitro-All Databases (webofscience.com) Impact of High-Molecular-Weight Hyaluronic Acid on Gene Expression in Rabbit Achilles Tenocytes In Vitro - PubMed (nih.gov)

## 1 Value of the Data

• Our data can be reused, if other cell types than tenocytes are stimulated with high-molecular weight HA *in vitro* – for a direct comparison of their doubling time and gene expression.
• These data can furthermore be reused, if tenocytes from other species than rabbits are stimulated with the same high-molecular weight HA *in vitro* to be compared to our data determined for rabbit tenocytes.
• These data can be reused for experimental comparison of rabbit Achilles tenocyte stimulation with other biomolecules than high-molecular weight HA, if the proliferation rate is of interest or if the same target gene expression of the cells is investigated.
• Our data could be further analyzed by correlating gene expressions of one target gene with another target gene and thus determine potential interdependency of target gene expression.
• Our data may help researchers in elucidating signalling mechanisms relating to proliferation, motility, inflammation, immunomodulation or other pathways, where hyaluronic acid plays a regulatory role.

## 2 Background

Besides fibrovascular scar formation [2], one major problem during tendon healing is adhesion formation to the surrounding tissue, particularly compromising intrasynovial flexor tendon healing. Therefore, implant materials with distinct anti-adhesive properties are designed to support and improve tendon healing by minimizing adhesion formation [3]. Hyaluronic acid (HA), a natural bio macromolecule, is largely present in the synovial fluid and is known to act as a very good lubricant, enabling unhindered gliding [4]. As the molecular weight of HA plays a pivotal role in differentially regulating several signaling pathways, we used a high-molecular weight HA for our *in vitro* monolayer tenocyte cell cultures. High-molecular weight HA (M_w_ > 10^6^ Da) is reported to be anti-angiogenic, anti-inflammatory and immunomodulating [5]. Hence, it might be interesting to know the effect of such HA on tenocytes in vitro. Therefore, data of cell proliferation and data of gene expression are presented, based on rabbit Achilles tenocyte exposure to 1.4 mg/mL high-molecular weight HA, and gene expression data were normalized to tenocyte cell cultures without HA.

## 3 Data Description

The data are stored as a Microsoft Excel (Microsoft Corporation, Redmond, WA, USA) file (.xlsx file) in two Mendeley Data repository services. Rabbit Achilles tenocytes proliferation: Alamar Blue® assay data for cell cultures with or without high molecular weight hyaluronic acid having a molecular weight range of 1.01–1.8 MDa - Mendeley Data and Impact of high-molecular weight hyaluronic acid on the gene expression of rabbit Achilles tenocytes in vitro - Mendeley Data.

The repository Rabbit Achilles tenocytes proliferation: Rabbit Achilles tenocytes proliferation: Alamar Blue® assay data for cell cultures with or without high molecular weight hyaluronic acid having a molecular weight range of 1.01–1.8 MDa - Mendeley Data contains one excel file, named Alamar Blue assay data HA.xlsx with two sheets described in detail under **Proliferation data**. The repository on gene expression Impact of high-molecular weight hyaluronic acid on the gene expression of rabbit Achilles tenocytes in vitro - Mendeley Data contains one excel file, named *qPCR tenocytes HA supplementation.xlsx* with 19 sheets; one for each target gene.

### 3.1 Proliferation data

**File** Alamar Blue assay data HA.xlsx

This excel file is open access published in Mendeley Data Rabbit Achilles tenocytes proliferation: Alamar Blue® assay data for cell cultures with or without high molecular weight hyaluronic acid having a molecular weight range of 1.01–1.8 MDa - Mendeley Data and contains 2 sheets, called *calibration curves rbTenocytes* and *HA-Exp R5, R8, R11*, respectively. The first sheet *calibration curves rbTenocytes* contains fluorescence intensities assessed for different rabbit Achilles tenocyte cell numbers per well (in line 7: cells / well), ranging from 500 to 128000 cells / well. Below, a log standard calibration curve is compared to the experimental calibration curve on the left side and for the whole range, while on the right side, a corresponding linear calibration is shown for the lower range of cells / well, from 500 to 32000 cells / well. In the second sheet entitled HA-Exp R5, R8, R11, the fluorescence intensities and the calculated cell numbers are shown, with all raw data for each of the three rabbits (R5, R8 and R11 are abbreviations for the three rabbit donors, R standing for rabbit and the number is the number of the rabbit from which the cells were harvested). Doubling times are calculated from line 38 downwards; and mean and standard deviation of doubling times are shown in columns I and J, respectively.

### 3.2 Gene expression data

**File** qPCR tenocytes HA supplementation.xlsx

This excel file is open access published in Mendeley Data Impact of high-molecular weight hyaluronic acid on the gene expression of rabbit Achilles tenocytes in vitro - Mendeley Data and contains 19 sheets, called *Biglycan, Decorin, Aggrecan, Collagen 1A1, Collagen 1A2, Collagen 3, LOX, IL-6, TNF-alpha, PAR-2, ALOX-15, Tenascin-C, Tenomodlin, Mohawk, alpha-SMA, TIMP-1, MMP-2, MMP-9* and *ki67*, respectively. These 19 sheets are arranged the same. In column A, the name of the target gene as well as the donor rabbit are given; where R5 means rabbit number 5, R8 rabbit number 8 and R11 rabbit number 11, respectively. In column B, the calculated 2^-ΔΔCT^ mean values are presented for time point 3 days without HA supplementation. In column C, the calculated 2^-ΔΔCT^ mean values are presented for time point 3 days with HA supplementation. In column D, the calculated 2^-ΔΔCT^ mean values are presented for time point 7 days without HA supplementation. In column E, the calculated 2^-ΔΔCT^ mean values are presented for time point 7 days with HA supplementation. In column F, the calculated 2^-ΔΔCT^ mean values are presented for time point 14 days without HA supplementation. In column G, the calculated 2^-ΔΔCT^ mean values are presented for time point 14 days with HA supplementation.

## 4 Experimental Design, Materials and Methods

### 4.1 In vitro cell culture using tenocytes harvested from rabbit Achilles tendons

Achilles tendons of female New Zealand White rabbits aged between 12 and 16 weeks served to harvest rabbit tenocytes (n = 3 different donors), and cell outgrowth/migration method was utilized (Veterinary license of Canton Zurich, reference number 255/15). To this aim, tendons were cut out immediately after euthanasia of the rabbits to ensure freshness and they were instantly washed with Hank's Balanced Salt Solution (1x HBSS with Ca^2+^ and Mg^2+^, Thermo Fisher Scientific, Rockford, IL, USA) with 200 µg/mL gentamycine (Biowest, Nuaillé, France) and with the antibiotic amphotericine B (Biowest, Nuaillé, France) in a concentration of 2.5 µg/mL according to [1]. The tissue around the tendons was carefully removed, and the middle area of all tendons was dissected into segments of less than 2 mm size and laved 3x with 1x HBSS buffer. After that, some tissue pieces were put into a sterile plate used for tissue cultures (PrimariaTM, Corning, New York, NY, USA) and approximately 50 µL of Ham's F12 (Biowest, Nuaillé, France), 10 % FBS (Biowest, Nuaillé, France), 200 µg/mL gentamycine, and 2.5 µg/mL amphotericine B) was dropped onto each piece. After leaving the tissues until they attached on the cell culture plates, which endured approximately 2 h in the incubator at 37 °C and 5 % CO_2,_ 10 mL of Ham's F12 were given to each plate.

We left the tissue culture plates with the tendon pieces like they were and did not agitate, rearrange nor handle them for approximately one week, in order to mitigate the risk of tendon tissue loosing adherence to the bottom of the plates and to promote cells migrating out from the tendon pieces without any disturbance. Cell culture medium was only exchanged five days after harvest. From then onwards, the culture medium was replaced on days 3, 6, 9 and 12 respectively. On day 14, the tendon tissue pieces were withdrawn from the plates, so only the cells were left in the plates. For another 7 days, cells could proliferate prior to preservation in liquid nitrogen at -196 °C. For expansion, such cryopreserved rabbit tenocytes were defrosted, resubmitted in Ham's F12 with 10 % FBS and 50 µg/mL gentamycine and cultured under standard conditions, where the temperature was 37 °C and the carbon dioxide concentration was 5 %. The culture medium was exchanged on days 2, 4, 6, etc. from then on until the desired cell number was obtained. To avoid a phenotypic drift typical for such tendon-derived cells at passages (P) higher than P4 [6], we used exclusively P2-4 for the proliferation and gene expression experiments from which data are described in the current data article.

### 4.2 Alamar Blue® assay to determine cell proliferation capacity

At time points 3, 5, 7 and 14 days, the metabolic activity and cell proliferation were measured using the Alamar Blue® assay (Invitrogen, # DAL1100, ThermoFisher Scientific, Basel, Switzerland). To do so, 350 cells/well were moved to a 96-well plate (TPP, # 92096, Trasadingen, Switzerland) using a volume of 100 µL Ham's F12 medium supplemented or not by 1.4 mg/mL HA manufactured using a microbial fermentation and purification process (Lifecore Biomedical, # HA 15M, Chaska, Minnesota, USA) and cultivated under standard conditions, i.e. 37 °C with 5 % CO_2_ (in technical triplicates). Culture medium containing HA was prepared and exchanged every day to avoid an extended decay of the long-chain HA [5], while medium without HA received an exchange only every other day. With a Cytation 5 imaging reader (BioTEk, Agilent Technologies AG, Basel, Switzerland), we determined the intensity at 530 nm (excitation) and at 590 nm (emission wavelength), after a 1/10 dilution of the Alamar Blue® assay solution in Ham's F12. Standard calibration curves were established in order to calculate the cell numbers belonging to specific fluorescence intensities according to the manufacturer's instruction of the Alamar Blue® assay. Calculation of doubling times (DT) was performed using the following equation, where *lg* denotes the logarithm for a basis of 10:

DT=Numberofdays×24h/(lgfactorofcellnumberincrease/lg2)

### 4.3 Gene expression of rabbit Achilles tenocytes

The tenocytes were cultured as described under *In vitro rabbit Achilles tenocyte cell culture using tenocytes harvested from rabbit Achilles tendons* and were transferred into 6-well plates (TPP, Trasadingen, Switzerland, growth area per well: 9.6 cm^2^) with an initial cell density of 2 × 10^5^cells in each well. The culture medium was changed to medium with HA (1. 4 mg/mL) or to reference medium without HA supplementation (Ham's F12, 10 % FBS, 50 µg/mL gentamycine) after one night during which the tenocytes settled and adhered to the ground of the wells. Importantly, the HA was added freshly to the medium at every medium exchange because it is known to decay and short-chain or low-molecular weight HA has different effects than high-molecular weight HA [5]. Cells from three different animals were used (n = 3 donors; denoted as R5, R8 and R11 in the Excel sheets of the corresponding repository Impact of high-molecular weight hyaluronic acid on the gene expression of rabbit Achilles tenocytes in vitro - Mendeley Data) where R is an abbreviation for *rabbit*), and experiments were performed in triplicates for each rabbit donor (n = 3) and furthermore with three technical replicates for each of these experiments (n = 3 of Real-time qPCR assessments); resulting in a total 27 data points per condition.

Utilizing the RNeasy Plus Mini Kit (Qiagen, Hilden, Germany, Switzerland) with RNase-free DNase (Qiagen, Hilden, Germany), total RNA was obtained at days 3, 7 or 14, according to the manufacturer's instructions. 500 ng of total RNA has been reverse-transcribed into cDNA within 20 µL of volume and the iScript Advanced cDNA Synthesis Kit was used (Bio-Rad, Cressier, Switzerland). To execute the PCR reactions, the corresponding cDNA samples were taken (5 ng cDNA per reaction), and the CFX Connect Real-Time PCR Detection System was applied (Bio-Rad, Cressier, Switzerland) as well as SsoAdvanced SYBR Green Supermix (Bio-Rad, Cressier, Switzerland). All PCR reactions had the following conditions: 95 °C for 3 min, with subsequent 39 cycles of 95 °C for 10 s and 62 °C for 30 s. This was repeated three times, leading to three technical replicates. All primer sequences are listed and can be found in Table S1 from the related research article [1]. They were purchased from Microsynth, Balgach, in Switzerland. Utilizing the 2^-ΔΔCT^ method for a comparative expression analysis [7], we calculated the relative gene expression of the chosen target marker genes. The results presented in the Excel files found under Impact of high-molecular weight hyaluronic acid on the gene expression of rabbit Achilles tenocytes in vitro - Mendeley Data are shown as manifold change normalized to the control, i.e. compared to samples cultivated without HA. The samples that did not contain HA were therefore set to 1 for all experiments.

## Limitations

Proliferation of rabbit tenocytes and gene expression data were only obtained for high-molecular weight HA, but not for low-molecular weight HA. Although low-molecular weight HA would therapeutically not be favourable, it would nevertheless be interesting to compare the gene response between high- and low-molecular weight HA supplementation to tenocyte cell cultures, particularly given the fact that high-molecular weight HA decays to low-molecular weight HA over time [5].

## Ethics Statement

The rabbit Achilles tenocytes were harvested from rabbits using an animal license approved by the veterinary office of Canton Zurich, Switzerland, having the reference number 255/15.

## Credit Author Statement

**Iris Miescher:** Conceptualization, Methodology, Data curation, Investigation, Writing – Original draft preparation, Writing – Reviewing and Editing. **Petra Wolint:** Data curation, Methodology, Investigation, Writing – Reviewing and Editing. **Christine Opelz:** Data curation, Writing – Reviewing and Editing. **Jess G. Snedeker:** Supervision, Writing – Reviewing and Editing. **Pietro Giovanoli:** Supervision, Writing – Reviewing and Editing. **Maurizio Calcagni:** Supervision, Writing – Reviewing and Editing. **Johanna Buschmann:** Conceptualization, Supervision, Methodology, Writing - Original draft preparation, Writing – Reviewing and Editing, Fund raising.

## Data Availability

Mendeley DataRabbit Achilles tenocytes proliferation: Alamar Blue® assay data for cell cultures with or without high molecular weight hyaluronic acid having a molecular weight range of 1.01-1.8 MDa (Original data). Mendeley DataImpact of high-molecular weight hyaluronic acid on the gene expression of rabbit Achilles tenocytes in vitro (Original data).
